# Integrative multi-omics identifies *MSR1* as a programmed cell death and extracellular matrix hub gene in osteoarthritis with hesperidin targeting potential

**DOI:** 10.3389/fimmu.2026.1777038

**Published:** 2026-05-21

**Authors:** Jinquan Bao, Lingling Wu, Wenqiang Zhao, Bo Li, Zilong Li, Zhigang Bai, Penglei Ma, Bo Gao, Chenggang Qiao

**Affiliations:** 1 The Second Affiliated Hospital of Inner Mongolia Medical University, Hohhot, Inner Mongolia Autonomous, China; 2 The Affiliated Hospital of Inner Mongolia Medical University, Hohhot, Inner Mongolia Autonomous, China

**Keywords:** extracellular matrix, hesperidin, immune microenvironment, molecular docking, MSR1, osteoarthritis, programmed cell death, WGCNA

## Abstract

**Background:**

Osteoarthritis (OA) is a degenerative joint disease with complex molecular mechanisms. There is an interplay between programmed cell death (PCD) and extracellular matrix (ECM) in inflammatory diseases. This study aimed to identify a key PCD/ECM-related gene involved in OA pathogenesis through multi-omics analysis.

**Methods:**

RNA-seq data of OA synovial tissue from the GEO databases were analyzed. Weighted gene co-expression network analysis (WGCNA), differential expression analysis, cross analysis, and protein-protein interaction (PPI) network were used to identify key PCD/ECM-related genes in OA. Regulatory networks were predicted using miRDB, TargetScan, and miRWalk databases, and transcription factor binding was analyzed using JASPAR and FIMO. The role of *MSR1* in OA progression was investigated by establishing an IL-1β-induced *in vitro* OA cell model.

**Results:**

We identified 517 genes associated with OA that were enriched in immune and developmental pathways. Differential expression analysis and cross-analysis revealed 24 OA-associated PCD- and ECM-related genes. *MSR1* was identified as the key candidate via PPI network analysis. In both the training and validation sets, *MSR1* expression was higher in OA samples than in healthy samples, with an AUC value greater than 0.7. *MSR1* expression was positively correlated with key immune populations and microenvironment scores, suggesting a role for *MSR1* in OA pathogenesis through immune modulation. In addition, molecular docking identified hesperidin as a high-affinity MSR1-binding compound with favorable interaction stability. *In vitro* experiments demonstrated that *MSR1* was upregulated in IL-1β–induced OA-like chondrocytes and promoted apoptosis, extracellular matrix degradation, and NF-κB pathway activation.

**Conclusion:**

This study highlights PCD/ECM-related gene *MSR1* as a central regulator of OA pathogenesis and proposes hesperidin as a potential therapeutic agent. The integrative approach provides a framework for understanding OA mechanisms and drug discovery.

## 1 Introduction

Osteoarthritis (OA) is a prevalent degenerative joint disorder characterized by progressive cartilage degradation, synovial inflammation, and subchondral bone remodeling, leading to chronic pain and functional impairment (1). According to statistics, the global number of OA cases reached about 375 million in 2021, with an age-standardized prevalence rate (ASPR) of 4,294.2 per 100,000 population (2). Projections indicate that the prevalence of OA will continue to rise worldwide by 2040, with estimated cases increasing from approximately 8.1 million to 16.6 million among men and from 11.3 million to 22.2 million among women (3). Despite its high global burden, the molecular mechanisms underlying OA pathogenesis remain incompletely understood, hampering the development of disease-modifying therapies. Emerging evidence suggests that dysregulated programmed cell death (PCD) pathways and extracellular matrix (ECM) disruption play pivotal roles in OA progression, with growing recognition of their crosstalk in inflammatory joint environments (4, 5).

PCD plays a pivotal role in the pathogenesis of OA, with apoptosis, pyroptosis, necroptosis, ferroptosis, and autophagy constituting the major contributing pathways. During advanced OA, chondrocyte apoptosis is markedly upregulated, which directly contributes to cartilage matrix degradation (6). Furthermore, pyroptosis exacerbates joint inflammation and cartilage destruction through NLRP3 inflammasome-mediated secretion of IL-1β and IL-18 (7). Importantly, these PCD pathways exhibit intricate crosstalk—accumulated apoptotic bodies can further trigger pyroptotic cascades, establishing a self-perpetuating cycle of chondrocyte loss (8), ultimately manifesting as characteristic OA pathologies including cartilage erosion and subchondral bone sclerosis (9). Moreover, necroptosis contributes to OA progression via oxidative stress and proinflammatory cytokine release (10), while ferroptosis manifests as GPX4 suppression and iron overload, accelerating chondrocyte demise (11, 12). Although autophagy serves as a cytoprotective mechanism by clearing damaged organelles, its dysregulation (e.g., aberrant mTOR signaling) paradoxically promotes OA pathogenesis (13, 14). The synergistic interplay of these PCD mechanisms orchestrates the core pathological cascade underlying cartilage degeneration in OA.

The ECM serves as the fundamental architectural framework of articular cartilage, providing both structural support and biomechanical cues for chondrocyte homeostasis. During OA progression, the dynamic equilibrium between ECM degradation and repair is profoundly disrupted, directly compromising cartilage integrity and function (15). Early OA is characterized by progressive ECM compositional alterations, where inflammatory mediators (e.g., IL-1β) and mechanical overloading converge to activate NF-κB and MAPK signaling cascades, resulting in elevated expression of matrix-degrading enzymes (MMPs and ADAMTSs) (16, 17). These proteolytic enzymes selectively cleave collagen fibers and proteoglycans, initiating irreversible ECM destruction that precedes macroscopic cartilage degeneration (16).

Emerging evidence reveals a reciprocal relationship between PCD and ECM breakdown in OA pathogenesis. Biomechanical stress induces chondrocyte apoptosis, which in turn exacerbates ECM degradation through the release of apoptotic bodies containing active MMPs (6, 18). The inflammatory microenvironment further amplifies this vicious cycle, as IL-1β simultaneously triggers both apoptotic pathways and pro-catabolic signaling, establishing a self-perpetuating “apoptosis-inflammation-matrix destruction” axis (19). Particularly noteworthy is the recently identified role of STING pathway activation in OA joints, where its upregulation orchestrates dual pathological effects: promoting ECM degradation while sensitizing chondrocytes to apoptosis (20). These findings highlight the synergistic mechanisms linking PCD and ECM degradation in OA. Therefore, a comprehensive analysis of the interplay between PCD and ECM in OA may provide crucial theoretical foundations for developing targeted therapeutic strategies.

In this study, we leveraged multi-omics datasets from public databases to identify a key gene involved in both PCD and ECM regulation in the pathogenesis of OA and further explored its potential functional role in the disease. Our findings advance the understanding of OA mechanisms while demonstrating a translatable framework for target discovery and drug repurposing in complex joint disorders.

## 2 Materials and methods

### 2.1 Data source

The RNA-seq data utilized in this study were obtained from publicly available OA synovial tissue datasets in the Gene Expression Omnibus (GEO) database, including GSE89408, GSE114007, GSE283079, GSE55235, and GSE46750. Detailed characteristics of each dataset are summarized in **Table 1**. To integrate datasets GSE89408, GSE114007, and GSE283079 derived from distinct platforms, raw count data from three independent public repositories were aggregated to construct a comprehensive training cohort comprising 51 healthy control and 78 OA synovial tissue samples. To mitigate technical batch effects introduced by heterogeneous sequencing platforms and experimental batches, the ComBat_seq function from the sva package (version 3.54.0) was applied, with dataset ID designated as the batch variable while preserving biological variation between disease groups (Healthy control vs. OA). Following batch effect correction, samples initially clustering by batch were effectively recentered, and inter-sample differences primarily reflected genuine biological heterogeneity (**Supplementary Figure 1**). The corrected count data were subsequently transformed to transcripts per million (TPM) and log2-transformed (log2[TPM + 1]) to generate a normalized expression matrix for downstream analyses. GSE55235 and GSE46750 served as independent validation sets.

**Table 1:** Information on all the datasets in this study.

Datasets	Osteoarthritis	Healthy	Experiment type	Platform
GSE89408	22	28	High throughput sequencing	GPL11154
GSE114007	20	18	High throughput sequencing	GPL11154
GSE283079	36	5	High throughput sequencing	GPL24676
GSE55235	10	10	Array	GPL96
GSE46750	12	12	Array	GPL10558

### 2.2 Identification of PCD- and ECM-related genes

Candidate genes associated with PCD and ECM were retrieved from the GeneCards database (v2022.11), which integrates multiple sources of evidence, including literature co-citation, functional annotation, and pathway enrichment, to generate a Relevance Score for each gene, with higher scores indicating stronger gene-function associations. Additionally, the GeneCards Inferred Functional Score (GIFtS) reflects the completeness of gene annotation, with higher scores representing better-characterized genes (21).

For PCD-related genes, the keyword “Programmed cell death” was used as the search term. Given the relative specificity of this keyword, which primarily yields genes with well-established roles in core cell death pathways, a higher Relevance Score threshold (>10) was applied to prioritize high-confidence core regulators while maintaining GIFtS > 50 to ensure functional annotation completeness. This strategy yielded 1,140 candidate PCD-related genes (**Supplementary Table 1**).

For ECM-related genes, the keyword “Extracellular matrix” was employed. This term encompasses a broad and heterogeneous family of genes, including structural components (e.g., collagens), proteases, integrins, and adhesion molecules. To avoid excluding functionally relevant genes due to the inherent diversity of this category, a more inclusive Relevance Score threshold (>3) was adopted, while the GIFtS > 50 criterion was retained to ensure annotation quality. This approach resulted in the identification of 2,272 ECM-related genes (**Supplementary Table 2**).

### 2.3 WGCNA

WGCNA was performed using the WGCNA R package (version 1.73) (22). To reduce technical noise, genes with low expression abundance (log2[TPM + 1] < 0.85) were filtered out before analysis. Pearson correlation coefficients were computed for all remaining gene pairs, and an optimal soft-thresholding power (β) was selected to satisfy scale-free topology criteria. A one-step network construction approach was employed to convert the adjacency matrix into a topological overlap matrix (TOM), followed by hierarchical clustering to generate a gene dendrogram. Gene significance and module eigengene-based significance measures were calculated to evaluate the biological relevance of identified modules. Inter-module relationships were analyzed, and intramodular gene lists were extracted for downstream functional characterization.

### 2.4 Differential gene expression analysis

Differential gene expression was analyzed using the “limma” R package (version 3.62.2) (23). Fold changes between OA and healthy control groups were calculated with subsequent multiple hypothesis testing correction using the false discovery rate (FDR) method. Differentially expressed genes (DEGs) were identified based on the |log2FC| > 1 and FDR < 0.05.

### 2.5 Functional enrichment analysis

Gene Ontology (GO) enrichment analysis (including Biological Process, Molecular Function, and Cellular Component categories) and KEGG pathway analysis were performed using the clusterProfiler R package (version 4.10.6) (24), with significantly enriched terms identified at an adjusted p-value threshold of <0.05. GO biological function analysis was further conducted using the ClueGO plugin (kappa score threshold >0.7, p-value<0.0001) in Cytoscape software (version 3.10.2).

For Gene Set Enrichment Analysis (GSEA), samples were dichotomized into high- and low-expression groups based on median gene expression levels. KEGG pathway enrichment was assessed between these groups, with significantly enriched pathways selected at FDR<0.05.

Gene Set Variation Analysis (GSVA) was implemented using the GSVA R package (version 2.0.6) to calculate pathway activity scores through a non-parametric, unsupervised approach. Differential pathway analysis between groups was subsequently performed using the limma package, with statistical significance assessed via t-tests and multiple testing correction using the FDR method (significance threshold: FDR<0.05).

### 2.6 Construction of protein-protein interaction (PPI) network

PPI networks were predicted using the STRING database (https://string-db.org/), with interaction confidence scores (combined_score) retained for subsequent analysis. The PPI network was constructed and visualized using Cytoscape software. Highly interconnected subnetworks were identified using the Molecular Complex Detection (MCODE) plugin, with default parameters applied to extract biologically relevant modules.

### 2.7 Construction of miRNA-mRNA regulatory network

The miRNA-mRNA regulatory network was constructed by integrating predictions from three independent databases: miRDB (https://mirdb.org/, binding score >80), TargetScan (https://www.targetscan.org/, percentile score >90), and miRWalk (http://mirwalk.umm.uni-heidelberg.de/, binding score >0.9), with only miRNAs consistently identified across all three databases being retained for subsequent analysis, followed by network visualization using Cytoscape software.

### 2.8 Prediction of transcription factor binding sites

Transcription factor binding sites were predicted using 1,518 transcription factor models corresponding to 656 genes from the JASPAR database (https://jaspar.genereg.net/; **Supplementary Table 3**), with genomic sequences spanning 2,000 bp upstream and 100 bp downstream of transcription start sites retrieved from UCSC (http://genome.ucsc.edu/). The FIMO online tool (https://meme-suite.org/meme/tools/fimo) was employed to identify potential transcription factor binding motifs in promoter regions, along with their specific binding locations and corresponding affinity scores, using position weight matrices obtained from JASPAR.

### 2.9 Immune microenvironment characterization

Single-sample gene set enrichment analysis (ssGSEA) is a widely adopted method in immuno-infiltration bioinformatics (25). Enrichment scores for immune cell subsets and immune-related functions were calculated for both OA and healthy control samples using the GSVA R package. Correlations between *MSR1* expression and the resulting scores were assessed via Spearman test. Additionally, the ESTIMATE algorithm (version 1.0.13) was employed to compute tumor microenvironment scores, including Immune, Stromal, and ESTIMATE scores.

### 2.10 Molecular docking

Molecular docking was performed using the three-dimensional structures of compounds obtained from PubChem (https://pubchem.ncbi.nlm.nih.gov) and the crystal structure of MSR1 protein retrieved from the RCSB-PDB database (https://www.rcsb.org/) [PDB ID: 7DPX], where compounds served as small molecule ligands and MSR1 as the receptor, with structural preprocessing including receptor dehydration and hydrogenation, along with ligand hydrogenation, conducted using Discovery Studio 4.5, followed by curvature-based cavity detection of five potential active pockets in MSR1 and molecular docking via the CB-DOCK2 server employing AutoDock Vina with FitDock methodology to identify the optimal binding pose, and subsequent two-dimensional and three-dimensional visualization of the complexes using Discovery Studio 4.5.

### 2.11 Molecular dynamics simulations

Molecular dynamics simulations were performed using GROMACS (version 2024.4) to evaluate protein-ligand interactions, initiated by system preparation through the application of CHARMM36 force field parameters and TIP3P water model to both receptor and ligand to generate topology files, followed by solvation in a periodic boundary box with ion addition for charge neutralization, where the simulation system was subsequently energy-minimized and equilibrated through NVT and NPT ensembles at 300 K and 1 bar respectively with continuous monitoring of temperature, pressure and density profiles, culminating in a production MD run of 10 ns duration, during which trajectory analyses including system re-centering and periodic boundary removal were performed, along with comprehensive evaluation of binding energetics, root-mean-square deviation (RMSD), root-mean-square fluctuation (RMSF) profiles, radius of gyration (Rg), and intermolecular hydrogen bond dynamics through detailed trajectory analysis.

### 2.12 Clinical samples

Between July 1 and September 10, 2025, synovial tissue samples were collected from 15 patients with OA and 15 healthy subjects (Normal control) in the Department of Joint Surgery and Sports Medicine at the Second Affiliated Hospital of Inner Mongolia Medical University. The mean age of the OA group was 69.07 ± 1.98 years, whereas that of the normal control group was 53.27 ± 11.10 years. Both cohorts comprised 4 males and 11 females. This study was approved by the Ethics Committee of Second Affiliated Hospital of Inner Mongolia Medical University (EFY20250115 (07)). All participants provided written informed consent before participation.

### 2.13 Cell line source and OA cell model construction

The immortalized chondrocytes (iCell-0092a) were purchased from iCell Bioscience Inc. (Shanghai, China) and maintained in DMEM/F12 medium supplemented with 1% penicillin-streptomycin (PS). The cells were cultured at 37 °C under a humidified atmosphere containing 5% CO_2_.

iCell-0092a cells were cultured in T25 flasks until reaching approximately 50% confluence. The cells were then trypsinized, seeded into a 96-well plate, and incubated for 24 hours at 37 °C under 5% CO_2_. Subsequently, the culture medium was replaced with DMEM/F12 complete medium containing 10 ng/mL IL-1β, and the cells were treated for 0, 6, 12, 24, and 48 h. Cell viability was assessed at each time point using a Cell Counting Kit-8 (CCK-8; C0039, Beyotime) according to the manufacturer’s instructions. Briefly, 10 μL of CCK-8 solution was added to each well, followed by incubation for 1 h at 37 °C. Absorbance was measured at 450 nm.

### 2.14 Cell transfection

Transfection was performed using a lipofectamine-based method. Briefly, iCell-0092a cells were transfected with siRNA (si-*MSR1*-1, -2, -3) or negative control (si-NC) using Lipofectamine 2000 in serum-free medium. After 48 h of transfection, cells were stimulated with IL-1β (10 ng/mL) for an additional 24 h to establish the OA-like cell model. The knockdown efficiency of *MSR1* was evaluated by RT-qPCR and Western blot analysis. The sequences of siRNAs and primers are listed in **Table 2**.

**Table 2:** Sequences of siRNAs used for *MSR1* knockdown.

siRNA designation	Sense strand	Antisense strand
si- *MSR1*-1	5’- GCGAAGAGGAAAUGAGAUU -3’	5’- AAUCUCAUUUCCUCUUCGC-3’
si- *MSR1*-1	5’- GCAGUUCUCAUCCCUCUCAUU-3’	5’- AAUGAGAGGGAUGAGAACUGC-3’
si- *MSR1*-1	5’- GCAACAUGGAGAAGAGAAU-3’	5’- AUUCUCUUCUCCAUGUUGC-3’

### 2.15 Quantitative real-time PCR

Total RNA was isolated from tissue or cells using TriQuick Reagent (R1100, Solaibao). RNA concentration and purity were determined with a NanoVue Plus spectrophotometer (111765, Healthcare Bio-Sciences AB). First-strand cDNA was synthesized from total RNA using the SureScript™ First-Strand cDNA Synthesis Kit (QP056T, GeneCopeia), according to the manufacturer’s protocol. The qRT-PCR assays were performed with the 2× SYBR Green qPCR Master Mix (Servicebio) on an iQ5 Real-Time PCR Detection System (G3320-05, Applied Biosystems). The PCR protocol was initiated at 95 °C for 3 min, followed by 40 cycles comprising 95 °C for 15 s and 60 °C for 30 s. The relative expression level of the target gene was calculated using the comparative 2^–ΔΔCt^ method. The sequences of the primers used for RT-qPCR are presented in **Table 3**.

**Table 3:** Primer sequences for RT-PCR.

Genes	Forward primer (5’-3’)	Reverse primer (5’-3’)
*MSR1*	CACTGATTGCCCTTTACCT	TTTCCCGTGAGACTTTGAG
*GM2A*	CGTGACATTAAGGAGAAGCTG	TAGAAGCATTTGCGGTGGAC

### 2.16 Western blot analysis

Total proteins were extracted using RIPA lysis buffer and quantified with a BCA assay kit. Protein samples were separated by 10% SDS-PAGE and transferred onto a PVDF membrane. After blocking, the membrane was incubated with the corresponding primary antibodies overnight at 4 °C, followed by incubation with an HRP-conjugated secondary antibody. Target proteins were detected using an enhanced chemiluminescence substrate, and band intensities were imaged and quantified with a chemiluminescence imaging system. The antibodies used in this study are shown in **Table 4**.

**Table 4:** Antibodies used in this study.

Antibody (Target)	Catalog/Clone No.	Manufacturer (Country)
Anti-GAPDH antibody	60004-1-Ig	Proteintech Group, Inc (China)
Anti-MSR1 antibody	ab123946	Abcam (UK)
Anti-β-actin antibody	66009-1-Ig	Proteintech Group, Inc (China)
Anti-Aggrecan antibody	A12045	ABclonal (China)
Anti-Collagen II antibody	A24334	ABclonal (China)
Anti-MMP13 antibody	A11148	ABclonal (China)
Anti-Phospho-NF-κB p65 antibody	AP1475	ABclonal (China)
Anti-NF-κB p65 antibody	A19653	ABclonal (China)
Anti-Bcl2 antibody	12789-1-AP	Proteintech Group, Inc (China)
Anti-Bax antibody	50599-2-Ig	Proteintech Group, Inc (China)
Anti-Cleaved caspase-3 antibody	25128-1-AP	Proteintech Group, Inc (China)

### 2.17 Flow cytometric analysis

Cell apoptosis was quantitatively assessed using flow cytometry following double-staining with fluorescein isothiocyanate (FITC)-labeled Annexin V and propidium iodide (PI). Briefly, harvested cells were washed once with phosphate-buffered saline (PBS) and resuspended in 1× binding buffer at a density of 1×10^6^ cells/mL. Subsequently, 100 μL of the cell suspension was transferred to a flow cytometry tube and incubated with 5 μL of Annexin V−FITC and 5 μL of PI solution in the dark for 15 min at room temperature. Afterwards, 400 μL of 1× binding buffer was added to each tube, and samples were analyzed by flow cytometry within 1 h. The percentages of apoptotic cells were determined by quantifying Annexin V^+^/PI^−^ (early apoptosis) and Annexin V^+^/PI^+^ (late apoptosis) subpopulations.

### 2.18 CETSA

iCell-0092a cells were pretreated with IL-1β (10 ng/mL) for 24 hours, followed by incubation with either Hesperidin (2 mg/mL) or DMSO for an additional 24 hours. The harvested cells were resuspended, aliquoted, and subjected to a temperature gradient ranging from 40, 43, 46, 49, 52, 55, 58, 61, 64, 67, and 70 °C for 3 minutes per condition using a metal bath, with a subsequent 3-minute equilibration at room temperature. Cells were lysed by repeated freeze-thaw cycles, and the soluble fraction was isolated by centrifugation at 12,000 rpm for 20 minutes at 4 °C. The supernatant was mixed with 2× SDS loading buffer, boiled for denaturation, and analyzed by Western blot to assess protein thermal stability. β-Actin was used as an internal control for quantification.

### 2.19 Statistical analyses

Statistical analyses were performed using R software (version 4.4.1), with intergroup gene expression differences assessed by Wilcoxon rank-sum tests, where a threshold of p < 0.05 was applied to determine statistical significance.

All experimental data were derived from three independent biological replicates and are presented as mean ± SD, with minimal variability confirmed among replicates. Statistical analyses were conducted using GraphPad Prism (version 9.4). Comparisons among multiple groups were conducted using one-way analysis of variance (ANOVA), followed by appropriate *post hoc* tests. A *p*-value < 0.05 was considered statistically significant.

## 3 Results

### 3.1 Identification of OA-associated gene modules through WGCNA

**Figure 1:**
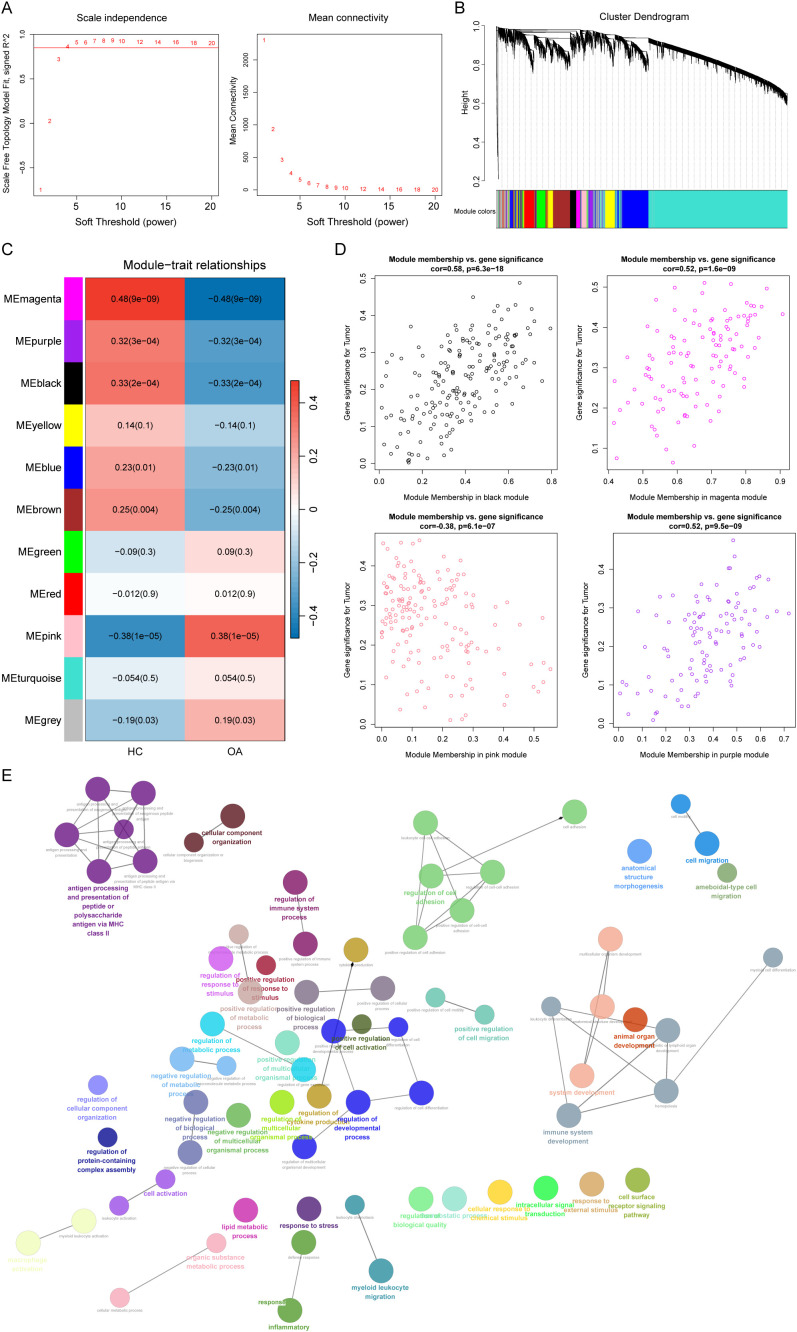
Identification of osteoarthritis (OA)-associated gene modules through WGCNA. **(A)** Analysis of the scale-free fitting index for soft threshold powers (β) and the mean connectivity for soft threshold powers. **(B)** The gene co-expression network and module results. **(C)** Module- OA/healthy sample correlation heatmap. **(D)** The scatter plot displays the correlation between module membership (x-axis) and gene significance for OA samples (y-axis). Each point represents a gene. **(E)** Functional enrichment analysis of 571 OA-associated genes.

To elucidate gene modules associated with the onset of OA, we performed WGCNA using samples from the training set. The optimal soft threshold was determined as β = 4 (**Figure 1A**), yielding 11 distinct gene modules (**Figure 1B**). Among these, four modules (magenta, purple, black, and pink) exhibited significant correlations with OA (**Figure 1C**). Further analysis revealed strong associations between module membership (MM) and gene significance (GS) for OA in these four modules (|correlation| > 0.3, p < 0.001; **Figure 1D**). Collectively, these modules encompassed 571 genes (**Supplementary Table 4**), which were designated as OA-related genes (OA-RGs). Functional enrichment analysis revealed that these genes were significantly enriched in OA-relevant biological processes and pathways, including ECM-receptor interaction, focal adhesion, and TNF signaling pathway (**Figure 1E**, **Supplementary Table 5**), all of which are well-established in OA pathogenesis. Notably, by capturing coordinated co-expression networks rather than relying solely on differential expression thresholds, WGCNA identified OA-associated modules that reflect disease-specific transcriptional programs.

### 3.2 Identification and functional characterization of OA-associated PCD and ECM-related genes

**Figure 2:**
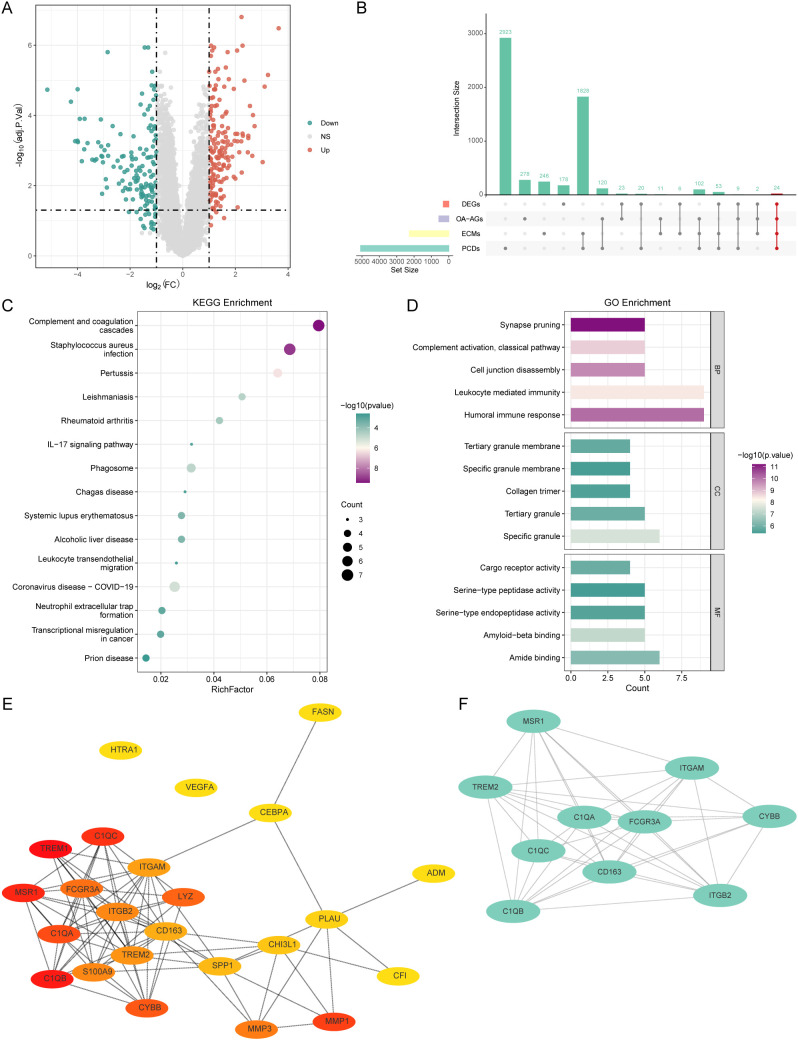
Identification and functional characterization of OA-associated PCD and ECM-related genes (OA- PCD/ECM). **(A)** Differentially expressed genes (DEGs) between OA and healthy groups in the training set. **(B)** Cross analysis among DEGs, programmed cell death (PCD)-related genes, extracellular matrix (ECM)-related genes, and OA-correlated genes (WGCNA). **(C)** The result of KEGG enrichment analysis. **(D)** The result of GO enrichment analysis. **(E)** PPI network of key genes. Node colors represent the degree of freedom of each gene within the network, while inter-node distances reflect correlation scores. **(F)** MCODE analysis in Cytoscape identified a highly clustered subnetwork.

Differential expression analysis between OA and healthy control groups in the training set revealed 315 significantly dysregulated genes (169 upregulated and 146 downregulated in OA (**Figure 2A**, **Supplementary Table 6**, FDR<0.05, |log2FC|>1). Intersection analysis of these DEGs with PCDs, ECMs, and OA-AGs identified 24 core hub genes potentially driving OA pathogenesis (**Figure 2B**). Functional enrichment analysis demonstrated these hub genes were highly enriched in 22 KEGG pathways (**Figure 2C**, **Supplementary Table 7**), such as complement and coagulation cascades, Staphylococcus aureus infection, and IL-17 signaling pathway, and 244 GO terms, such as humoral immune response, cell junction disassembly, and Amyloid-beta binding (**Figure 2D**, **Supplementary Table 7**).

### 3.3 Identification of *MSR1* as a key OA-PCD/ECM gene

**Figure 3:**
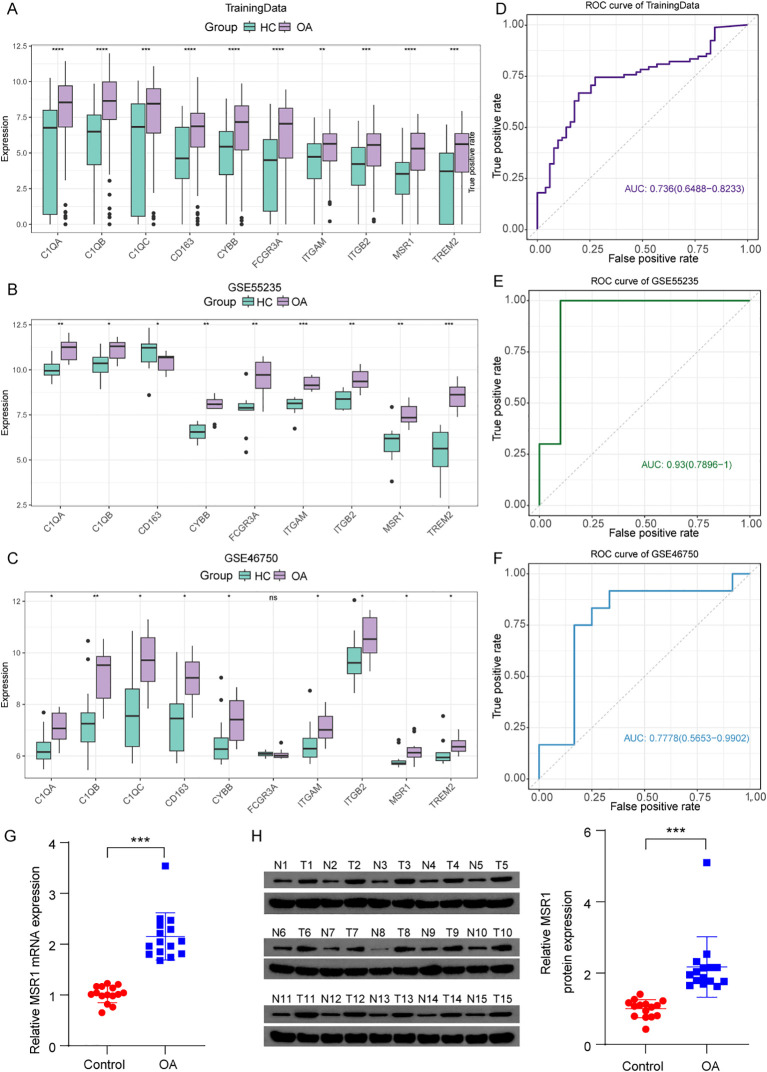
The expression patterns and diagnostic value of *MSR1*. **(A–C)** The expression of genes in OA and healthy groups in the training set and validation sets (GSE55235 and GSE46750). **(D–F)** The receiver operating characteristic (ROC) curve of *MSR1* in the training set and validation sets (GSE55235 and GSE46750). **(G)** The expression of *MSR1* mRNA levels in clinical synovial tissue from OA patients and healthy volunteers. **(H)** The expression of MSR1 protein levels in clinical synovial tissue from OA patients and healthy volunteers. *p < 0.05, **p < 0.01, ***p < 0.001, ****p < 0.0001; ns, not significant.

To identify core OA-PCD/ECM genes, we constructed a PPI network using the STRING database, which comprised 24 nodes and 83 edges with an average node degree of 3 (**Figure 2E**). Edge lengths were weighted by interaction scores, and node colors reflected connectivity. Subsequent MCODE analysis in Cytoscape identified a highly clustered subnetwork (score = 9.3) containing ten genes (**Figure 2F**). Next, we analyzed the expression of these ten genes in OA and normal control samples across the training set and two validation cohorts (GSE55235 and GSE46750). As shown in **Figures 3A–C**, the expression levels of *C1QA*, *C1QB*, *CYBB*, *ITGAM*, *ITGB2*, *MSR1*, and *TREML2* were significantly higher in the OA group compared to the healthy controls in all three datasets. Receiver operating characteristic (ROC) curve analysis further revealed the diagnostic potential of *MSR1* for OA, yielding AUC values exceeding 0.7 in the training set (0.736) and both validation sets (GSE55235: 0.930; GSE46750: 0.778) (**Supplementary Table 8**; **Figures 3D–F**). Based on these findings, *MSR1* was selected as the key gene for subsequent analyses. To further validate these findings, we examined *MSR1* expression in synovial tissue samples obtained from 15 OA patients and 15 healthy controls using RT-qPCR and western blotting. Consistent with the bioinformatic analysis, both mRNA and protein levels of MSR1 were significantly elevated in the clinical OA synovial tissue samples compared to healthy controls (**Figures 3G, H**).

### 3.4 Potential functional information of *MSR1* in OA

**Figure 4:**
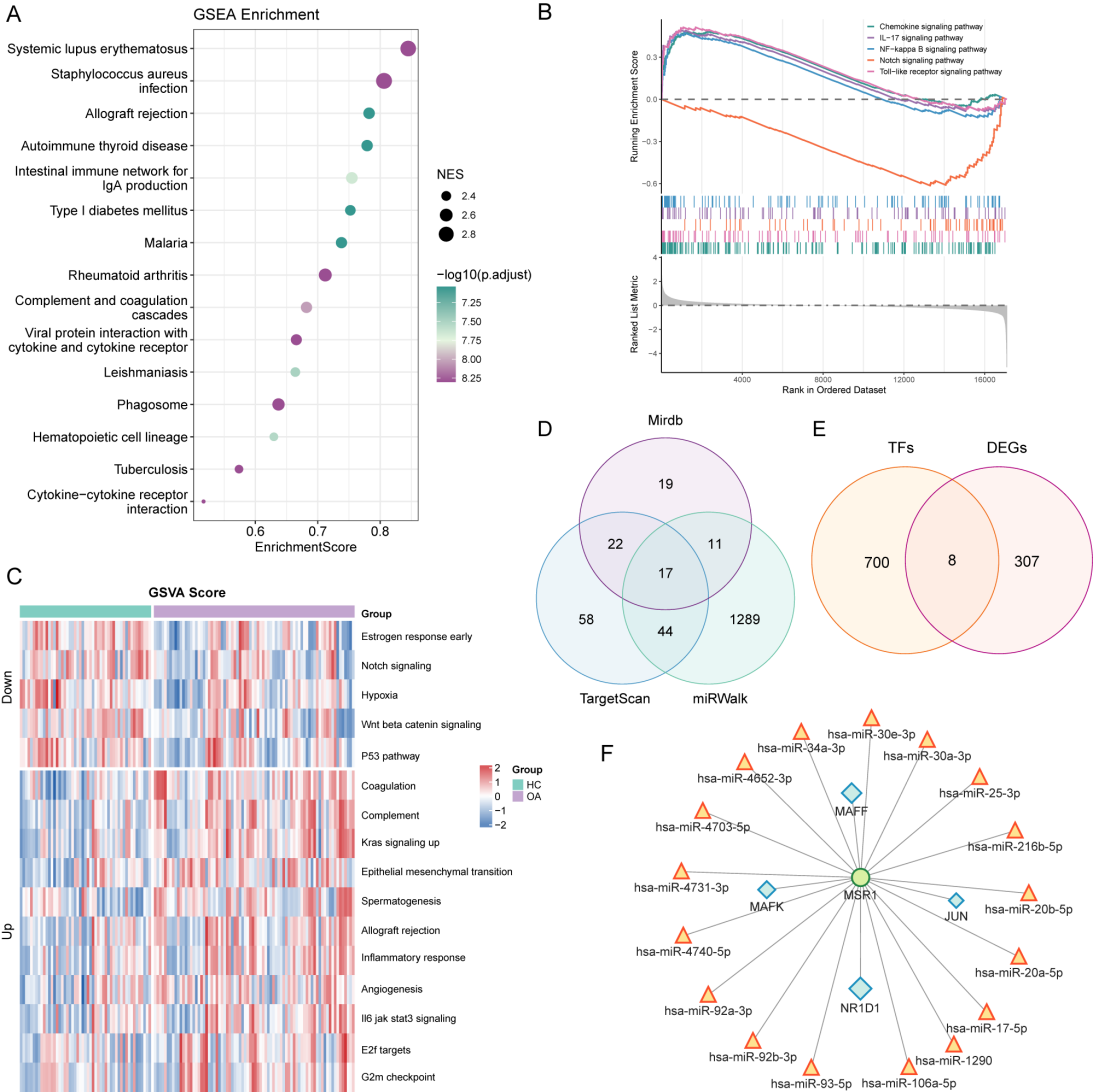
Potential functional information, predicting miRNA and TF of *MSR1* in OA. **(A)** Bubble plot displaying the top 15 enriched pathways from GSEA analysis of differentially expressed genes between *MSR1*-high and *MSR1*-low expression groups. The x-axis represents the gene enrichment score (NES), the y-axis shows pathway names, bubble color intensity corresponds to -log10(P-value), and bubble size reflects the normalized enrichment score (NES). **(B)** Chemokine signaling pathway, IL−17 signaling pathway, NF−kappa B signaling pathway, and Notch signaling pathway were significantly activated in the *MSR1*-high group. **(C)** Heatmap displaying GSVA enrichment scores of significantly differentially activated pathways between *MSR1*-high and *MSR1*-low groups. **(D)** Venn diagram showing intersecting miRNAs predicted by three independent databases (miRDB, TargetScan, and miRWalk). **(E)** Venn diagram illustrating the intersection between differentially expressed transcription factors (TFs) between OA and healthy groups and known TF gene sets from the FIMO online tool. **(F)** miRNA-transcription factor-mRNA regulatory network.

In the training set, OA samples were stratified into *MSR1*-high and *MSR1*-low groups based on median *MSR1* expression levels. Subsequently, DEGs between the OA and healthy control groups were identified. To elucidate the functional relevance of *MSR1* in OA pathogenesis, GSEA and GSVA were performed on these DEGs within the training set. The GSEA results indicated that these DEGs between the *MSR1*-high and *MSR1*-low groups were significantly enriched in the Chemokine signaling pathway, IL−17 signaling pathway, NF−kappa B signaling pathway, and Notch signaling pathway (**Figures 4A, B**, **Supplementary Table 9**). Additionally, GSVA revealed a correlation between the *MSR1* and Kras signaling up, Notch signaling, and Wnt beta-catenin signaling (**Figure 4C**, **Supplementary Table 9**).

### 3.5 Predicting miRNA and TF of *MSR1*

To investigate *MSR1*’s transcriptional regulation in OA, we first identified 17 putative *MSR1*-targeting miRNAs by cross-referencing predictions from three databases (miRDB, TargetScan, and miRWalk) (**Figure 4D**, **Supplementary Table 10**). Moreover, we identified 315 differentially expressed transcription factors (TFs) between OA and healthy control groups. We subsequently employed the FIMO online tool to systematically evaluate the binding capacity of these 315 TFs to the *MSR1* promoter region (**Figure 4E**). Using a stringent significance threshold (p-value < 0.0001), we confirmed that four TFs (JUN, MAFF, MAFK, and NR1D1) exhibited specific binding to the *MSR1* promoter (**Supplementary Table 11**). Finally, based on these computational predictions, we constructed a comprehensive miRNA-TF-*MSR1* regulatory network using Cytoscape, which visually delineates the multi-layer regulatory relationships potentially governing *MSR1* expression in OA (**Figure 4F**). It is important to note, however, that these predicted interactions remain hypothetical and warrant experimental substantiation in subsequent investigations.

### 3.6 *MSR1* modulates the immune microenvironment in OA

**Figure 5:**
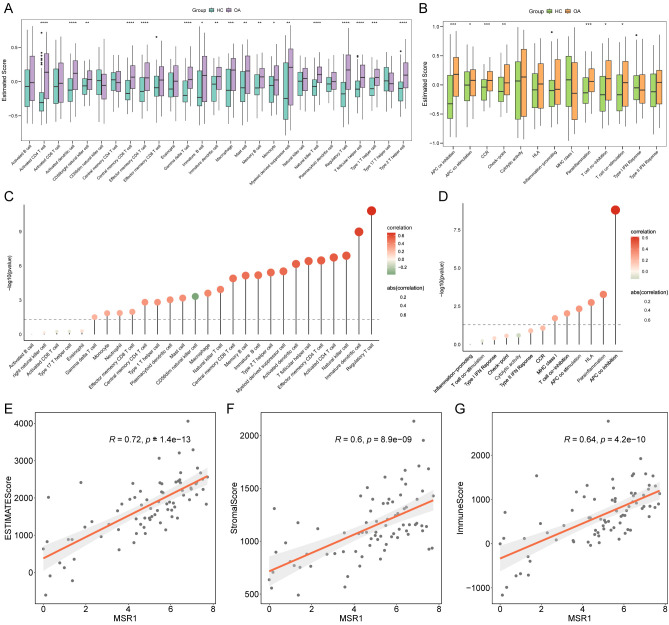
*MSR1* modulates the immune microenvironment in OA. **(A, B)** ssGSEA analysis on the training set to comprehensively evaluate the infiltration levels of 28 immune cell subsets and the activity of 13 immune-related functions in both OA and healthy groups. **(C, D)**. The correlation of *MSR1* expression with immune cell subsets and immune-related functions. **(E–G)** The correlation of *MSR1* expression with ESTIMATEScore, StromalScore, and ImmuneScore. *p<0.05, **p<0.01, ***p<0.001, ****p<0.0001.

To investigate the role of *MSR1* in regulating the immune microenvironment of OA, we first performed ssGSEA analysis on the training set to comprehensively evaluate the infiltration levels of 28 immune cell subsets and the activity of 13 immune-related functions in both OA and healthy groups (**Figures 5A, B**). Notably, OA patients exhibited significantly elevated infiltration of 18 immune cell types (including Activated CD4+T cells, Regulatory T cells, Central memory CD8+T cells, and Effector memory CD4+T cells) along with enhanced enrichment scores for 7 immune functions (such as APC co-inhibition, CCR, and Parainflammation) compared to the healthy group (p<0.05) (**Figures 5A, B**). Subsequent correlation analysis revealed that *MSR1* expression levels in OA patients were positively associated with several immune cell populations (e.g., Regulatory T cells (Tregs), Immature dendritic cells, and natural killer (NK) cells; p<0.01) and the APC co-inhibition function (**Figures 5C, D**). Furthermore, *MSR1* expression was significantly correlated with ESTIMATEScore, StromalScore, and ImmuneScore (**Figures 5E–G**), suggesting that *MSR1* may contribute to OA pathogenesis by modulating the immune microenvironment.

### 3.7 Identification of hesperidin as a potent MSR1-binding compound from dandelion flavonoids

**Figure 6:**
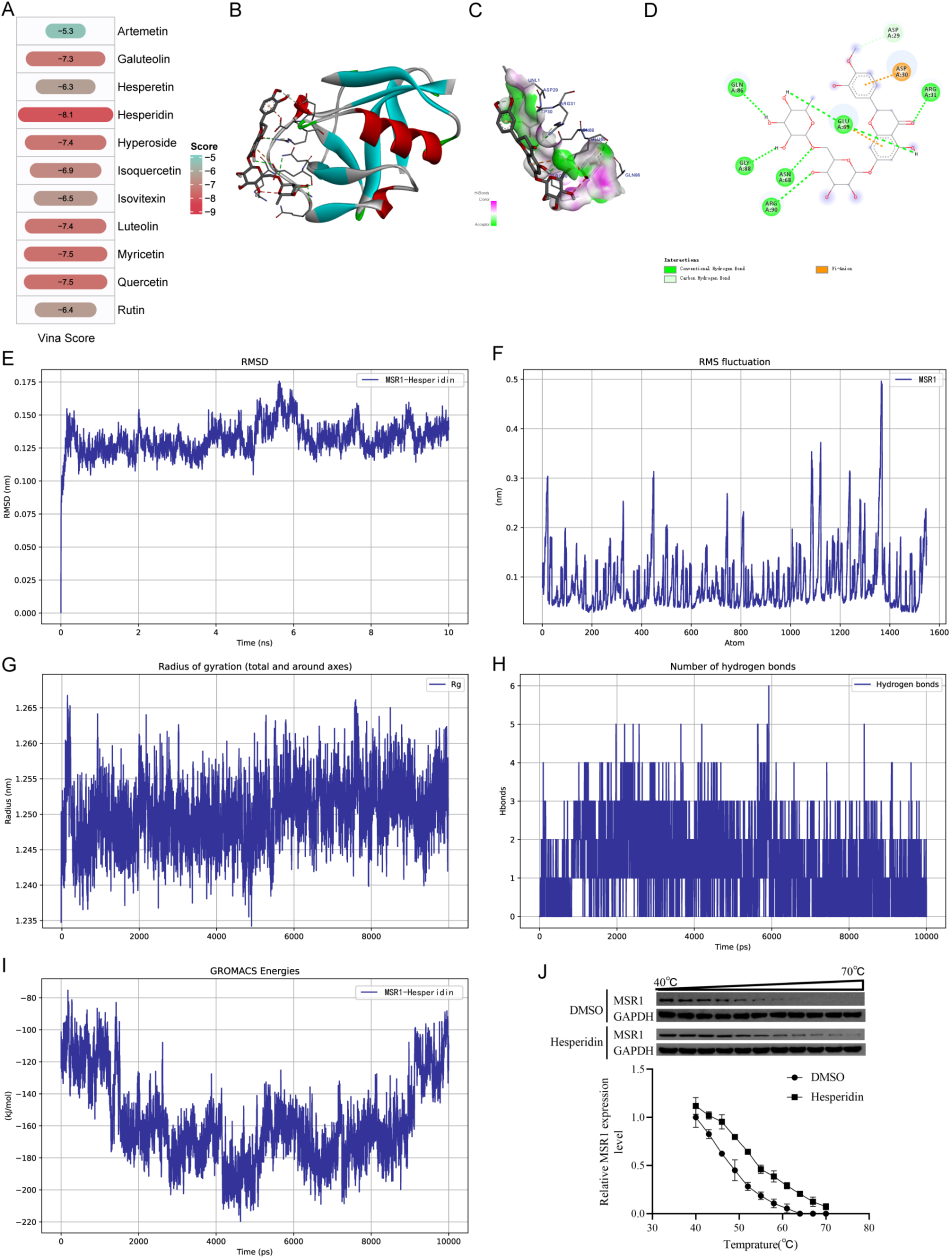
Identification of Hesperidin as a Potent MSR1-Binding Compound from Dandelion Flavonoids. **(A)** Molecular docking score heatmap. Both tile size and color gradient represent binding affinity scores. **(B)** 3D structural representation of the MSR1-Hesperidin binding mode. **(C)** Crystallographic structure of hesperidin bound to the active pocket of MSR1. **(D)** 2D interaction diagram of hesperidin with MSR1 protein residues. **(E)** Root-mean-square deviation (RMSD) trajectory plot. **(F)** Root-mean-square fluctuation (RMSF) profile plot. **(G)** Radius of gyration (Rg) variation plot. **(H)** Protein-ligand hydrogen bond count plot. **(I)** Total system energy trajectory plot. **(J)** CETSA experiment evaluated the thermal stability of the MSR1-hesperidin complex.

Systematic screening of the TCMID database (https://bidd.group/TCMID/) identified 65 bioactive constituents from dandelion, including 11 flavonoid compounds (**Supplementary Table 12**). Molecular docking analysis revealed that hesperidin exhibited the strongest binding affinity to MSR1 protein with a docking score of -8.1 kcal/mol (**Figure 6A**, **Supplementary Table 12**). Structural characterization demonstrated that hesperidin precisely occupies the active pocket of MSR1 formed by critical residues (ASP29, ASP30, ARG31, ASN68, GLU69, GLN86, GLY88, and ARG90), forming seven hydrogen bonds (**Figures 6B–D**). To evaluate the dynamic stability of the MSR1-hesperidin complex, we performed 10-ns molecular dynamics simulations (**Supplementary Figure 2**). The system reached equilibrium after 1 ns, with the RMSD of the protein backbone maintained at 0.12-0.15 nm (fluctuation < 0.05 nm) and an average RMSD of 0.13 nm (**Figure 6E**). RMSF analysis indicated overall structural stability with limited residual fluctuations (**Figure 6F**). Secondary structure analysis confirmed the maintenance of stable protein folding (**Figure 6G**), with three persistent hydrogen bonds and two dynamic hydrogen bonds observed throughout the simulation (**Figure 6H**). Energy profiling showed the system reached its lowest energy state (-220 kJ/mol) at 5 ns (**Figure 6I**). Moreover, the CETSA results demonstrated that hesperidin binding significantly stabilized MSR1 in the OA cell model, with a pronounced shift in its melting curve observed between 40 and 70 °C (**Figure 6J**).

### 3.8 MSR1 promotes IL-1β−induced OA progression by regulating apoptosis, ECM metabolism, and NF−κB signaling

**Figure 7:**
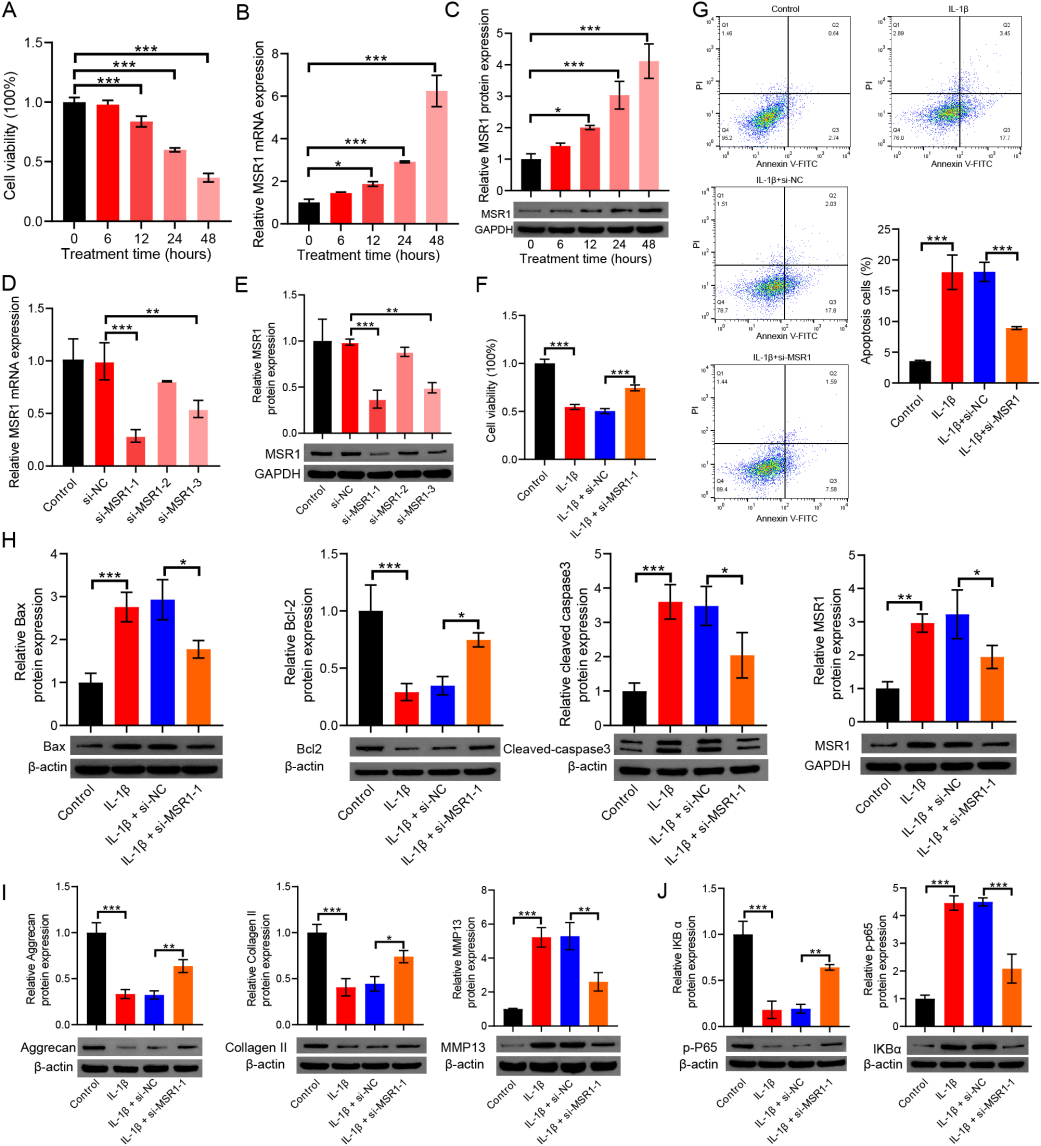
*MSR1* is upregulated in IL-1β–induced OA-like chondrocytes and promotes apoptosis, extracellular matrix degradation, and NF-κB pathway activation. **(A)** Cell viability of iCell−0092a chondrocytes treated with 10 ng/mL IL−1β at different time points was assessed by CCK−8 assay. B−C. *MSR1* expression at mRNA **(B)** and protein **(C)** levels after IL−1β treatment for the indicated durations. D−E. Validation of *MSR1* knockdown efficiency by RT−qPCR **(D)** and Western blot **(E)**. **(F)** Cell viability after *MSR1* knockdown in IL−1β–treated cells. **(G)** Apoptosis rate measured by flow cytometry following *MSR1* silencing under IL−1β stimulation. **(H)** Expression levels of *MSR1* and apoptosis−related proteins (cleaved−caspase 3, Bax, and Bcl−2) detected by Western blot in the different experimental groups. **(I)** Protein expression of ECM−related markers (collagen II, aggrecan, and MMP13) in the different experimental groups. **(J)** Effects of *MSR1* knockdown on NF−κB signaling, shown by protein levels of IκBα and phosphorylated p65 (p−p65). Data are presented as mean ± SD from three independent experiments. *p < 0.05, **p < 0.01, ***p < 0.001.

To investigate the potential role of *MSR1* in OA, we conducted a series of *in vitro* experiments. First, the CCK−8 assay was used to evaluate the impact of IL−1β (10 ng/mL) treatment on the viability of iCell−0092a cells across different time points. A 24−h treatment period was identified as optimal for establishing an OA−like cell model, based on its significant effect on cell viability (**Figure 7A**). Using this model, we observed that both mRNA and protein expression levels of MSR1 were progressively upregulated with prolonged IL−1β stimulation, peaking significantly at 24 h and 48 h (**Figures 7B, C**). Therefore, iCell−0092a cells treated with 10 ng/mL IL−1β for 24 h were employed as the OA model in all subsequent experiments.

To further elucidate the functional contribution of *MSR1* in OA progression, we established an *MSR1*−knockdown system in iCell−0092a cells. RT−qPCR and Western blot analyses confirmed that both si−*MSR1*−1 and si−*MSR1*−3 effectively suppressed *MSR1* expression compared with the si−NC group, with si−*MSR1*−1 demonstrating higher knockdown efficiency (**Figures 7D, E**). Next, the *MSR1*-knockdown cells were stimulated with IL-1β (10 ng/mL) for 24 h to establish the OA-like cell mode. As shown in **Figure 7F**, *MSR1* knockdown notably reversed the IL−1β−induced reduction in cell viability. Flow cytometric analysis further revealed that *MSR1* silencing significantly attenuated IL−1β−triggered apoptosis (**Figure 7G**).

Consistently, *MSR1* knockdown downregulated the expression of pro−apoptotic proteins, cleaved−caspase 3, and Bax, while upregulating the anti−apoptotic protein Bcl−2 (**Figure 7H**). We next examined the effect of *MSR1* knockdown on ECM metabolism. As shown in **Figure 7I**, compared with the IL−1β + si−NC group, the IL−1β + si−*MSR1*−1 group exhibited significantly elevated levels of key ECM−synthetic proteins (collagen II and aggrecan), whereas the expression of the matrix−degrading enzyme MMP13 was markedly suppressed.

Based on GSEA results indicating significant activation of the NF−κB signaling pathway in the *MSR1*−high group, we further explored the regulatory role of *MSR1* in this pathway. Western blot analysis demonstrated that *MSR1* knockdown substantially increased the protein level of IκBα and reduced the phosphorylation of p65 under IL−1β stimulation (**Figure 7J**), suggesting that *MSR1* may participate in OA progression by modulating the NF−κB pathway.

## 4 Discussion

OA is a chronic disease characterized primarily by the degeneration of articular cartilage, with its pathological progression involving multiple complex mechanisms. Among these, PCD and ECM degradation are two pivotal processes that interact closely and collectively drive OA progression. Apoptotic, pyroptotic, and ferroptotic chondrocytes release inflammatory factors and matrix-degrading enzymes, further accelerating ECM destruction (26–28). Conversely, ECM disruption alters the chondrocyte microenvironment, leading to cellular stress and death. For instance, ECM degradation activates the NF-κB pathway, thereby promoting inflammation and apoptosis (26, 29). In this study, we employed bioinformatics analysis to identify a key PCD/ECM-related gene, *MSR1*, implicated in OA pathogenesis. *MSR1* expression was significantly upregulated in OA samples compared to healthy controls, with an AUC value exceeding 0.7, suggesting its potential as a diagnostic biomarker. Furthermore, *MSR1* exhibited strong correlations with pro-inflammatory signaling pathways and immune cell infiltration in OA.

*MSR1* is a pattern recognition receptor (PRR) predominantly expressed in myeloid cells. It plays a crucial role in maintaining immune homeostasis (30) and is implicated in the pathogenesis of diverse diseases, including inflammatory disorders (31), cancers (32), neurodegenerative diseases (33), infectious diseases (34), and metabolic diseases (35). Notably, *MSR1* promotes neuronal apoptosis by activating the NF-κB signaling pathway and inducing the release of inflammatory mediators in the presence of myelin debris (36). Additionally, *MSR1* serves as a positive regulator of autophagy during bacterial infection, where its activation induces autophagic clearance of intracellular pathogens via the PI3K-Akt-mTOR pathway (37). Regarding ECM biology, MSR1 promotes macrophage adhesion to the extracellular matrix via proteoglycan binding (38) and contributes to ECM remodeling through MMP secretion in tumor-associated macrophages (39). Beyond its functional roles, *MSR1* has emerged as a promising diagnostic biomarker across several pathologies. Elevated expression of *MSR1*-positive cells correlates with disease progression and poor post-lung transplant prognosis in idiopathic pulmonary fibrosis (IPF) (40). Similarly, MSR1 expression is significantly upregulated in monocytes from patients with acute coronary syndrome (ACS) and associated with disease severity (41). In the present study, we observed significant upregulation of *MSR1* in OA samples, with an AUC value exceeding 0.7. In the IL-1β-induced OA cell model, we observed that *MSR1* expression was significantly upregulated. Functionally, *MSR1* knockdown attenuated the IL-1β-induced reduction in cell viability, suppressed apoptosis, and promoted ECM accumulation. These findings identify *MSR1* as a dual-functional gene involved in both ECM remodeling and programmed cell death, positioning it as a promising diagnostic biomarker for OA. Although direct evidence linking *MSR1* to OA pathogenesis remains limited, it is noteworthy that *MSR1* plays a pivotal role in bone metabolism and regeneration. For example, during fracture healing, *MSR1* mediates macrophage uptake of fatty acids (FAs) from apoptotic chondrocytes, activating PPARα and promoting FAO (42). This metabolic reprogramming enhances macrophage osteoinductive capacity via BMP7 upregulation and NAD^+^/SIRT1/EZH2 axis activation (42). Furthermore, *MSR1* promotes osteogenic differentiation of bone marrow-derived mesenchymal stem cells (BMSCs) by activating the PI3K/AKT/GSK3β/β-catenin signaling pathway (43). Given that OA involves metabolic dysregulation and impaired repair mechanisms in joint tissues, including subchondral bone, the observed upregulation of *MSR1* in OA, coupled with its established critical functions in bone metabolism, collectively suggests that *MSR1* may contribute not only as a diagnostic marker but also potentially to the pathological processes or repair responses underlying OA.

Functional enrichment analysis revealed that inflammation-related signaling pathways (IL-17, NF-κB) were enriched in OA patients with elevated *MSR1* expression. IL-17—a pleiotropic inflammatory cytokine primarily secreted by Th17 cells—is known to be involved in articular cartilage degradation and synovitis during OA pathogenesis (44). Mechanistically, IL-17 can activate the NF-κB pathway, thereby inducing the release of inflammatory mediators including IL-1β, IL-6, and matrix metalloproteinases (MMPs), which are associated with cartilage destruction and synovial inflammation (44, 45). Furthermore, IL-17 has been linked to OA progression through the upregulation of cellular senescence markers such as p16INK4a and p21 (45). Notably, high *MSR1* expression was positively correlated with increased proportions of regulatory Tregs and NK cells within the OA microenvironment. Tregs are known to suppress inflammatory responses and promote tissue repair through secretion of anti-inflammatory cytokines such as IL-10 and TGF-β (46, 47). Their expansion in OA may reflect an adaptive immunoregulatory mechanism associated with efforts to counteract excessive inflammation and immunopathology (48). In this context, Tregs have been shown to attenuate the IL-17/NF-κB inflammatory axis by inhibiting Th17 cell activity and IL-17 production (48). Concurrently, NK cells may contribute to the resolution of inflammatory stimuli by enhancing neutrophil antimicrobial function via GM-CSF secretion (49). Moreover, we found that *MSR1* knockdown inhibited NF-κB signaling by stabilizing IκBα and attenuating p65 phosphorylation, suggesting that *MSR1* promotes OA progression through activation of this pro-inflammatory pathway. Collectively, these findings position *MSR1* as a key molecular node in OA pathogenesis, where it integrates pro-inflammatory signaling (NF-κB) with the regulation of chondrocyte survival, ECM homeostasis, and adaptive immune responses, suggesting that targeting *MSR1* may offer multiple therapeutic benefits.

Several limitations of this study should be acknowledged. First, the selection of PCD- and ECM-related genes from GeneCards based on predefined thresholds may introduce annotation bias and exclude under-characterized but relevant genes. Complementary database integration or experimental validation would help refine these gene sets. Second, the proposed miRNA-TF-MSR1 regulatory network is prediction-based and requires experimental confirmation. Third, although the iCell-0092a cell line maintains key chondrocytic properties and enables reproducible mechanistic studies, it may not fully reflect the complexity of primary human chondrocytes; validation using primary cells or *in vivo* OA models is warranted. Fourth, the CCK-8 assay primarily measures metabolic activity rather than directly assessing cell survival, so the observed anti-apoptotic effects following MSR1 knockdown should be interpreted with caution and confirmed by complementary viability assays. Fifth, while multi-omics analysis identified MSR1 as a key gene bridging multiple programmed cell death pathways in OA, functional validation in this study focused solely on apoptosis. Whether MSR1 regulates pyroptosis or ferroptosis in OA remains to be elucidated and warrants further investigation.

## 5 Conclusion

In conclusion, our study identifies the PCD/ECM-associated gene *MSR1* as a key pathogenic driver in OA and nominates hesperidin as a promising therapeutic candidate. Mechanistically, *MSR1* exacerbates OA progression under inflammatory stress by coordinately enhancing chondrocyte apoptosis, disrupting ECM homeostasis, and activating the NF-κB signaling pathway. The present findings provide important mechanistic insights into the function of *MSR*1 in chondrocytes; however, future validation using primary human chondrocytes or cartilage explant cultures is warranted to enhance the clinical relevance of our conclusions.

## Data Availability

The original contributions presented in the study are included in the article/**Supplementary Material**. Further inquiries can be directed to the corresponding author.

## Supplementary material

Supplementary Figure 1 Principal component analysis (PCA) plots with or without the elimination of batch effects. **(A)** PCA plot without batch effect elimination. **(B)** PCA plot with batch effect elimination. **(C)** The expression levels of samples without the elimination of batch effects in the training set. **(D)** The expression levels of samples with the elimination of batch effects in the training set.

Supplementary Figure 2 Molecular dynamics simulations. **(A)** Potential energy variation diagram of the simulated system. **(B)** Temperature variation diagram of the simulated system. **(C)** Pressure variation diagram of the simulated system. **(D)** Density variation diagram of the simulated system.

Supplementary Table 1 The list of programmed cell death (PCD)-related genes.

Supplementary Table 2 The list of extracellular matrix (ECM)-related genes.

Supplementary Table 3 The list of transcription factors.

Supplementary Table 4 Osteoarthritis (OA)-associated genes identified through Weighted Gene Co-expression Network Analysis (WGCNA).

Supplementary Table 5 Results of ClueGO enrichment analysis.

Supplementary Table 6 Differentially expressed genes between OA and healthy groups.

Supplementary Table 7 Functional enrichment analysis results of GO and KEGG pathways.

Supplementary Table 8 Area under the curve (AUC) values of *MSR1* for OA diagnosis in the training and validation cohorts.

Supplementary Table 9 Enrichment analysis outcomes from GSEA and GSVA analysis.

Supplementary Table 10 miRNA prediction results integrated from three databases (miRDB, TargetScan, and miRWalk).

Supplementary Table 11 Transcription factor binding analysis results.

Supplementary Table 12 Lists of active compounds and flavonoids from Taraxacum spp.
