# Supplementary figures and images for: Integrative multi-omics identifies MSR1 as a programmed cell death and extracellular matrix hub gene in osteoarthritis with hesperidin targeting potential
Source: Front Immunol. 2026 May 21;17:1777038. doi: 10.3389/fimmu.2026.1777038 (PMC13233285; doi:10.3389/fimmu.2026.1777038)

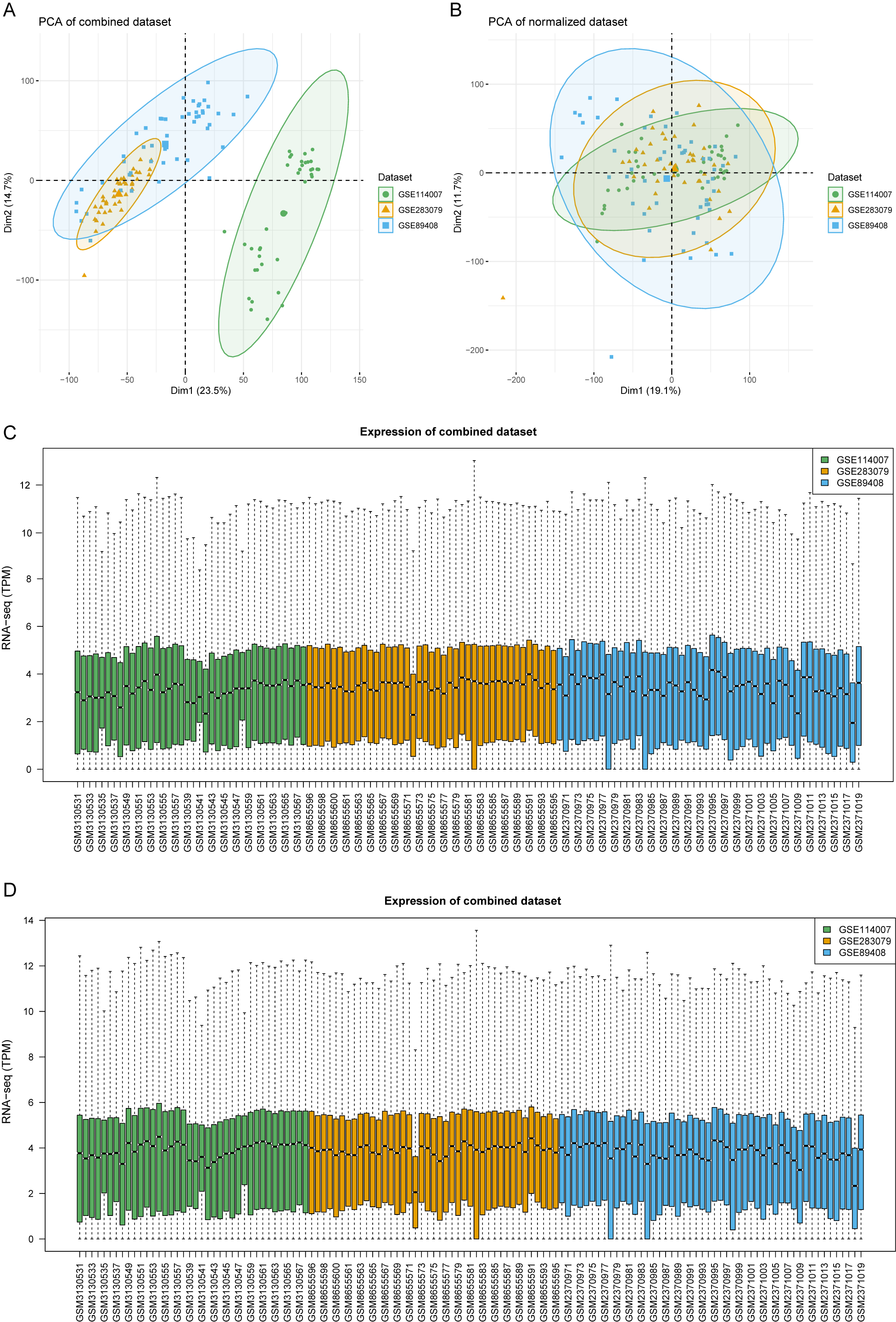

Supplement: Supplementary Figure 1 — Principal component analysis (PCA) plots with or without the elimination of batch effects. (A) PCA plot without batch effect elimination. (B) PCA plot with batch effect elimination. (C) The expression levels of samples without the elimination of batch effects in the training set. (D) The expression levels of samples with the elimination of batch effects in the training set. [file Image1.tif]

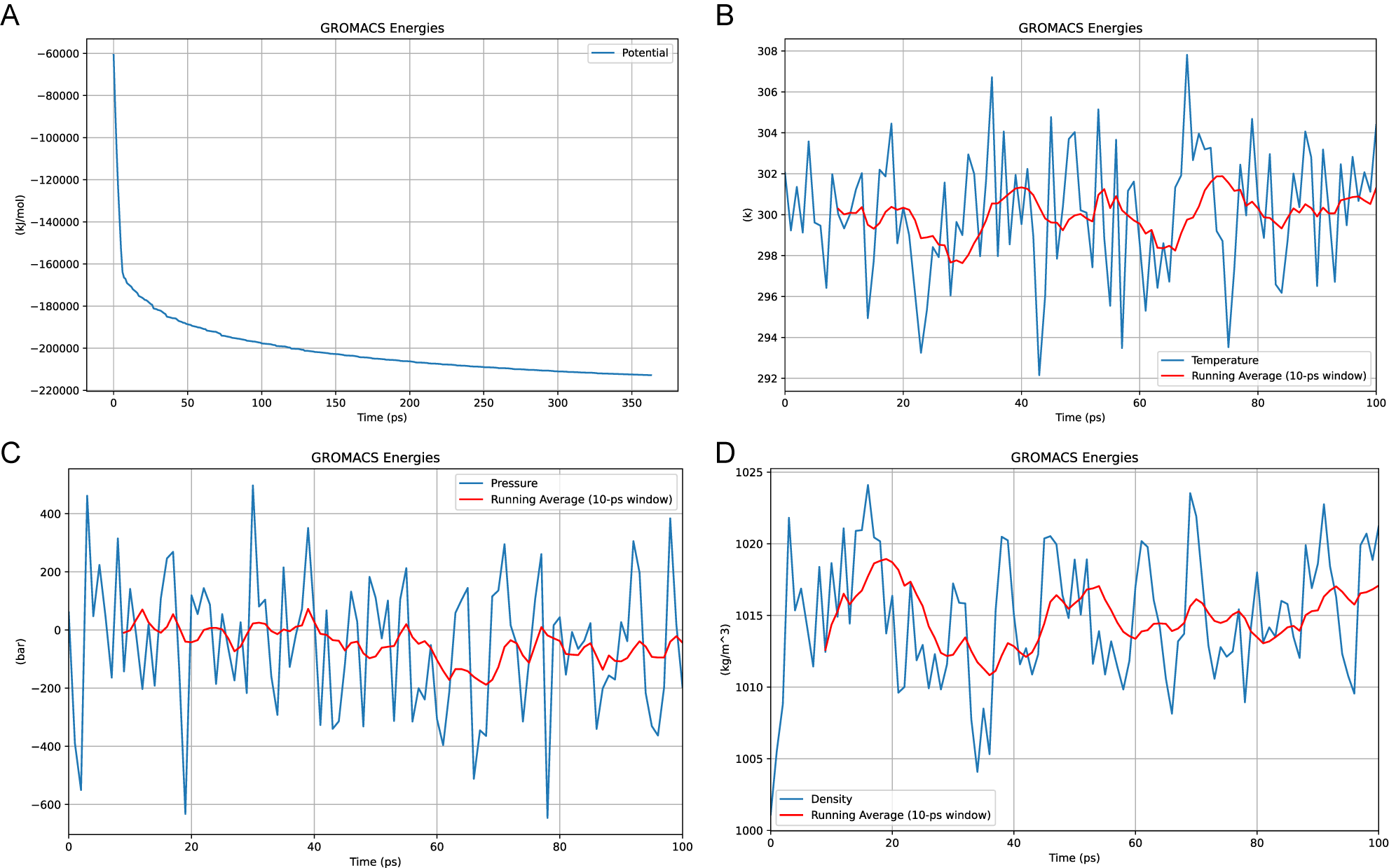

Supplement: Supplementary Figure 2 — Molecular dynamics simulations. (A) Potential energy variation diagram of the simulated system. (B) Temperature variation diagram of the simulated system. (C) Pressure variation diagram of the simulated system. (D) Density variation diagram of the simulated system. [file Image2.tif]
